# Morphine Decreases Enteric Neuron Excitability via Inhibition of Sodium Channels

**DOI:** 10.1371/journal.pone.0045251

**Published:** 2012-09-21

**Authors:** Tricia H. Smith, John R. Grider, William L. Dewey, Hamid I. Akbarali

**Affiliations:** 1 Department of Pharmacology and Toxicology, Virginia Commonwealth University, Richmond, Virginia, United States of America; 2 Department of Physiology and Biophysics, Virginia Commonwealth University, Richmond, Virginia, United States of America; Temple University School of Medicine, United States of America

## Abstract

Gastrointestinal peristalsis is significantly dependent on the enteric nervous system. Constipation due to reduced peristalsis is a major side-effect of morphine, which limits the chronic usefulness of this excellent pain reliever in man. The ionic basis for the inhibition of enteric neuron excitability by morphine is not well characterized as previous studies have mainly utilized microelectrode recordings from whole mount myenteric plexus preparations in guinea pigs. Here we have developed a Swiss-Webster mouse myenteric neuron culture and examined their electrophysiological properties by patch-clamp techniques and determined the mechanism for morphine-induced decrease in neuronal excitability. Isolated neurons in culture were confirmed by immunostaining with pan-neuronal marker, β-III tubulin and two populations were identified by calbindin and calretinin staining. Distinct neuronal populations were further identified based on the presence and absence of an afterhyperpolarization (AHP). Cells with AHP expressed greater density of sodium currents. Morphine (3 µM) significantly reduced the amplitude of the action potential, increased the threshold for spike generation but did not alter the resting membrane potential. The decrease in excitability resulted from inhibition of sodium currents. In the presence of morphine, the steady-state voltage dependence of Na channels was shifted to the left with almost 50% of channels unavailable for activation from hyperpolarized potentials. During prolonged exposure to morphine (two hours), action potentials recovered, indicative of the development of tolerance in single enteric neurons. These results demonstrate the feasibility of isolating mouse myenteric neurons and establish sodium channel inhibition as a mechanism for morphine-induced decrease in neuronal excitability.

## Introduction

The enteric nervous system (ENS) extends the length of the gastrointestinal tract and regulates digestive functions including peristalsis, secretion, sensation of stimuli, blood flow etc.; for review see [1]. This ‘second brain’ is unique in that it can reflexively function without input from the central nervous system, and contains more neurons and neurotransmitters than anywhere else in the peripheral nervous system [2].

Two major classes of neurons have been extensively characterized in the myenteric plexus of the guinea pig ENS: afterhyperpolarization (AHP) neurons, also referred to as AH neurons, and synaptic (S) neurons [3]–[5], alternatively called Dogiel types II and I, respectively; for review see [6]. AHP neurons are so named for their large afterhyperpolarization following an action potential [3]. Morphologically, these neurons have multiple long projections [7] and are characterized as sensory neurons [8], [9]. S type neurons feature only one long axon and are either motor neurons or interneurons [10]. Furthermore, AHP and S neurons exhibit distinct immunoreactivity; for review, see [11]. AHP neurons stain positively for calbindin [4], [7], [12], while S neurons show positive calretinin staining [12] and calcitonin gene-related peptide (CGRP) staining within the myenteric plexus. [13], [14].

Morphine and other opioids directly affect the ENS, causing severe constipation through reduced peristalsis, increased water and electrolyte absorption, and antisecretory actions [15]. The exact molecular mechanisms for these effects are unclear. It is well established that morphine acutely decreases neurotransmitter release via a pre- and post-synaptic mechanism [16]–[19], and that morphine can decrease the firing rate of myenteric neurons [20].

Previous studies examining the effects of opioids on enteric neurons have largely been limited to sharp intracellular microelectrode recordings in guinea pigs, which limits the studies to examine voltage changes without insight into the biophysical properties of individual ion channels. Reduced neuronal excitability in the presence of morphine has been attributed to hyperpolarization caused by increased K^+^ conductance [21]–[23]. The hyperpolarizations were transient in nature, often declining progressively after 2 to 3 minutes, and did not occur in all neurons tested. The specific ionic conductances affected are unclear.

In the present study we have developed methodology for primary culture of adult mouse myenteric neurons, examined the electrophysiological properties via whole cell patch clamp technique and determined the effect of morphine on neuronal excitability and ionic currents. We distinguished two subtypes of enteric neurons in culture and found that morphine suppresses excitability of neurons with AHPs due to inhibition of TTX-insensitive sodium currents. These findings suggest a novel mechanism for morphine–mediated inhibition of enteric neuron excitability.

## Methods

### 2.1 Isolation and culture of cells from adult mouse myenteric plexus

#### Ethics Statement

All animal care and experimental procedures were in accordance with and approved by the Institutional Animal Care and Use Committee at Virginia Commonwealth University.

All chemicals and reagents were obtained from Sigma Aldrich (St Louis, MO), unless otherwise noted, except cell culture reagents, which were purchased from Gibco (Grand Island, NY). Male Swiss Webster mice (26–30 g, Harlan Sprague Dawley, Inc.) were killed by cervical dislocation. The ileum was immediately dissected and placed in ice-cold Krebs solution (in mM: 118 NaCl, 4.6 KCl, 1.3 NaH_2_PO_4_, 1.2 MgSO_4_ 25 NaHCO_3_, 11 glucose and 2.5 CaCl_2_) bubbled with carbogen (95% O_2_/5% CO_2_). Ileal segments were threaded longitudinally on a plastic rod through the lumen and the longitudinal muscle with the myenteric plexus (LMMP) was gently removed using a cotton-tipped applicator. LMMP strips were rinsed three times in 1 ml Krebs and gathered by centrifugation (350× *g*, 30 s). LMMP strips were then minced with scissors and digested in 1.3 mg/ml collagenase type II (Worthington) and 0.3 mg/ml bovine serum albumin in bubbled Krebs (37°C) for 1 hour, followed by 0.05% trypsin for 7 min. Following each digestion, cells were triturated and collected by centrifuge (350× *g* for 8 min). Cells were then plated on laminin (BD Biosciences) and poly-D-lysine coated coverslips in Neurobasal A media containing B-27 supplement, 1% fetal bovine serum, 10 ng/ml glial cell line-derived neurotrophic factor (GDNF, Neuromics, Edina, MN), and antibiotic/antimycotic liquid. Half of the cell media was changed every 2–3 days.

### 2.2 Immunohistochemistry/Immunocytochemistry

Whole mount preparations of mouse ileal segments were carried out on LMMP tissues. LMMP segments were placed on coverslips with outer longitudinal muscle side facing downward. The remaining segment of ileum was removed from the plastic rod, cut open longitudinally, and pinned mucosal side up in a dissecting dish filled with bubbled Krebs solution. The mucosa/submucosa was gently removed and the remaining circular muscle transferred to a coverslip with the side previously attached to mucosa faced down. Whole mounts were fixed in 4% formaldehyde for 2 hours. Isolated cells cultured for 7–10 days on coverslips were fixed in 4% formaldehyde for 30 min. Cells and tissues were permeabilized with Triton X-100 (0.05% tissue, 0.01% cells) in PBS (30 min), and blocked with 10% goat serum (1 hr). Preparations were incubated with the primary antibody overnight at 4°C. Primary antibodies used were as follows: Neural specific anti-βIII-tubulin (rabbit, Abcam ab18207-100, 1∶100), anti-glial fibrillary acidic protein (GFAP, mouse, Chemicon MAB360, 1∶500), anti-calbindin (rabbit, Chemicon AB1778, 1∶1,000) anti-calretinin (rabbit, Swant CR 7699/3H, 1∶2,000), anti-μ opioid receptor (rabbit, Immunostar 24216, 1∶1,000) and anti-μ opioid receptor (rabbit, Neuromics RA10104, 1∶100),

Following 3 washes in PBS, cells were incubated with the appropriate secondary antibodies; either goat anti-rabbit or goat anti-mouse Alexa 488 Dye (Molecular Probes, 1∶1,000, 1 hr, RT). Nuclei were stained with Hoechst 33342 dye (1 µg/ml). Visualization was performed on an Olympus Fluoview Confocal Microscope and software (v5.0).

### 2.3 Electrophysiology

Neuronal cells were studied from 1–4 days after plating on coverslips. Cells were placed in an experimental chamber and perfused (1–2 ml/min) with an external physiological solution containing (in mM): 135 NaCl, 5.4 KCl, 0.3 NaH_2_PO_4_, 5 HEPES, 1 MgCl_2_, 2 CaCl_2_, and 5 glucose. Patch electrodes (2–3 MΩ) were pulled from borosilicate glass capillaries (Sutter Instruments, CA) and filled with internal solution containing (in mM): 100 K-aspartic acid, 30 KCl, 4.5 ATP, 1 MgCl_2_, 10 HEPES, and 0.1 EGTA. In some experiments, the internal solution was substituted with Cs^+^ containing solution (in mM): 100 L-aspartic acid, 30 CsCl, 1 MgCl_2_, 5 HEPES, 2 ATP, 5 EGTA, 0.1 GTP. Whole-cell patch-clamp recordings were made with an Axopatch 200B amplifier (Molecular Devices, CA) at room temperature and pulse generation and data acquisition were achieved with Clampex and Clampfit 10.2 software (Molecuar Devices, CA). Initial cell characteristics were determined in current clamp mode with current provided in 13 sweeps of 0.5 s duration ranging from −0.03 nA to 0.09 nA in 0.01 nA increments. Current-voltage relationships were determined in voltage clamp mode, in 16 0.5 s-sweeps beginning at −100 mV and increasing in 10 mV intervals to 50 mV. Current amplitudes were normalized to cell capacitance (pF) to determine current density. Action potential (AP) derivatives were determined using the differential function in Clampfit software. The threshold of APs were determined as the voltage at which the d(V)/d(T) function deviated from zero. AP height was determined by measuring the threshold to the peak of the AP. Voltage-dependence of steady-state inactivation and activation were determined and fit via Boltzman distribution as described previously [24].

### 2.4 Data Analysis

Results are presented as mean ± SEM for the number of cells (n). Statistical tests were performed using GraphPad Prism 5.0 software using Matched Two-way ANOVA or two-tailed t-test. Values of p<0.05 were regarded as significant.

## Results

### 3.1 Immunohistochemistry of LMMP and Cultured LMMP cells

**Figure 1:**
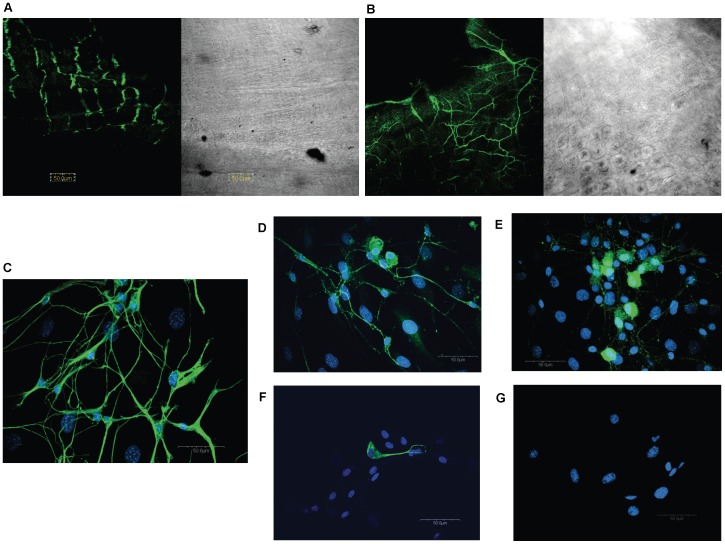
Immunohistochemical characterization of enteric neurons in the mouse. Confocal microscopy revealed neuronal-specific βIII-tubulin (Abcam, rabbit, 1∶1,000) staining in whole mount ileal longitudinal muscle (A) and circular muscle (B) preparations from the mouse. Cells isolated from longitudinal muscle/myenteric plexus (LMMP) preparations contain neurons (C; βIII-tubulin, Abcam, rabbit, 1∶1,000) that stain postiviely for calbindin (D) (Chemicon, rabbit, 1∶1,000) and calretinin (E) (Swant, rabbit, 1∶2,000). Glia (F) were visualized with the glia-specific marker GFAP (Chemicon, mouse, 1∶500). Antibodies were visualized via appropriate goat secondary antibody Alexa 488 (green, Molecular Probes, 1∶1,000)0. Nuclei were visualized using Hoescht 33342 (blue, C–G, 1 µg/ml). No staining was seen when primary antibody was omitted (G).

Upon peeling off the longitudinal muscle layer, myenteric neurons were identified on both the longitudinal and circular muscle preparations in whole mount immunohistochemistry using the βIII tubulin antibody (Fig. 1A & 1B).

Isolated enteric neurons were prepared from longitudinal muscle myenteric plexus (LMMP) preparations as this provided better access to myenteric neurons without contamination. They were placed on glass coverslips coated with poly-D-lysine and laminin. Neurons attached to coverslips after one day in culture and long neuronal projections were evident after 2 days. Other rounder, flatter cell types were also observed. After a week in culture, some neurons formed clusters that appeared to form a ganglionic-like plexus over a layer of the flatter, wider cells present in the culture that may be glia. Mouse myenteric neurons stained positively for the neuron-specific marker βIII tubulin (Fig. 1C).and some stained additionally for the calcium-binding proteins, calretinin (Fig. 1D) or calbindin (Fig. 1E). Glia were also identified in the culture by GFAP (Fig. 1F). Cell nuclei were identified by co-staining with the Hoechst 3342 fluorescent DNA dye (Fig. 1 C–G). Immunoreactivity was not detected in the absence of primary antibodies (Fig. 1G).

Two different antibodies were used to detect the μ opioid receptor and both demonstrated non-specific staining. The anti- μ opioid receptor antibody by Immunostar showed a high level of staining throughout the cell both before and after pre-absorption with the immunizing μ opioid receptor peptide (data not shown). The Neuromics μ opioid receptor antibody showed membrane-localized staining in both wild-type Swiss Webster mice and μ opioid receptor-knock out C57 mice (data not shown). Thus, similar to other reports [25] we found that primary antibodies for the μ-opioid receptors are highly non-specific.

### 3.2 Whole Cell Patch Clamp Characterization of Cultured Enteric Neurons

Whole cell patch clamp recordings were performed on more than 100 mouse myenteric neurons. Initial characterization examined 33 neurons using a current clamp protocol of 13 sweeps beginning with a current injection of −0.03 nA for 200 ms, and increasing stepwise by 0.01 pA to 0.09 pA with a 15 s start-to-start interval. In voltage clamp protocol, the cells were clamped at Vh = −60 mV and then depolarized for 300 ms from −100 mV to +50 mV in 10 mV steps.

**Figure 2:**
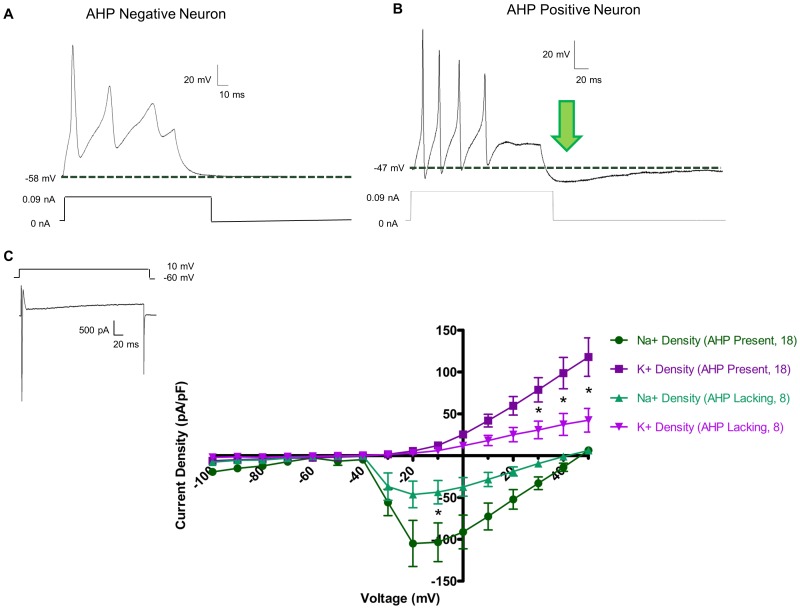
Cultured myenteric neurons are comprised of two electrophysiologically distinct populations. In current clamp mode (A & B), all neurons displayed action potentials upon current injection of 0.09 nA. Upon cessation of current stimulation, neurons either returned to their original resting membrane potential (A), or dipped below baseline in a slow after-hyperpolarization (AHP, arrow, B). AHPs have an average magnitude of −7.41±0.98 mV, a duration of 212.27±24.98 ms, and a τ = 98.47±13.12 ms. In voltage clamp mode (C), sharp inward spikes of Na^+^ current followed by a sustained outward K^+^ current were readily apparent (inset). Current density – voltage relationships of Na+ and K+ currents in both AHP negative and AHP positive neurons showed that AHP positive had significantly greater current densities as determined by two-way ANOVA (* = p<0.05).

**Figure 3:**
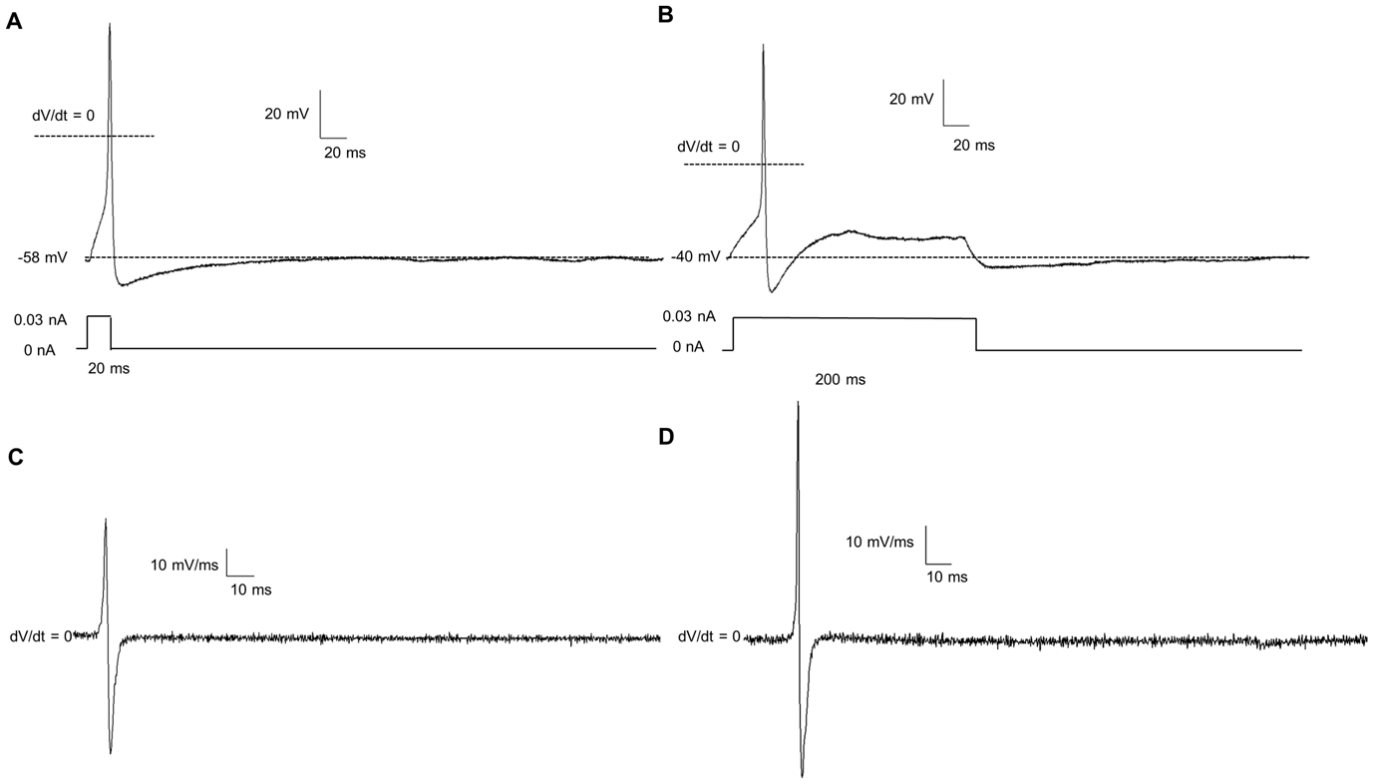
Short and long current stimuli result in APs followed by an after-hyperpolarization (AHP). Both short (20 ms, A) and long (200 ms, B) current injections (0.03 nA) result in an immediate AHP following the action potential and after the stimulus is removed. The derivative of the action potential (dV/dt) for short (C) and long (D) stimuli show no evidence of a “hump” on the repolarization phase of the action potential.

Neurons were delineated based on their *sine qua non*, or defining feature, the action potential (AP; Fig. 3). In addition, neurons were also characterized by the presence/absence of an afterhyperpolarization (AHP; Fig. 2 A & B). Initially, both subtypes of neurons fired a single AP which progressed to multiple APs with increasing current strength. A majority of both neuron types fired in a “phasic” manner at maximum current injection (0.09 nA) with multiple spikes followed by a plateau. In some cells from both subtypes, multiple action potentials were present during the stimulus (0.5 s) corresponding to “tonic” –like neurons. The threshold, the amount of current which initially generated an AP, was not different between cell types defined as AHP positive (+ve) and AHP negative (−ve) (0.031±0.008 nA in AHP +ve neurons vs. 0.029±0.007 nA in AHP-ve neurons, Table 1). Action potentials were initiated at the same voltage (determined via dV/dt) in AHP +ve and AHP −ve neurons (−31.1±2.4 mV in AHP +ve vs. −25.6±2.1 mv in AHP −ve neurons). The height of the action potential was not different between AHP +ve and −ve neurons (74.3±5.0 mV in −ve neurons vs. 69.8±7.2 mV in AHP +ve neurons). The duration of the AP (measured at 30% of the AP height) did not differ between the two subtypes (2.50±0.28 ms in AHP +ve neurons vs. 3.08±0.65 ms in −ve neurons). We also examined neurons at shorter current durations (20 ms). In the AP examples shown in Figure 3 A & B, a prominent AHP was observed at the end of the stimulus. The derivatives of the action potentials, dV/dt (Fig. 3 C & D), showed that there was no significant hump in the downward phase of the action potential. The resting membrane potential did not significantly differ between the two cell types (−49.6±1.6 mV in AHP +ve neurons vs. −51.8±2.2 mV in AHP −ve neurons).

**Table 1:** Electrophysiological Characteristics of AHP positive and AHP negative enteric neurons.

	AHP +ve Neurons (21 Neurons)	AHP −ve Neurons (12 Neurons)
Cell Capacitance (pF)	23.24±3.16	16.08±1.973
Resting Membrane Potential (mV)	−49.6±1.6	−51.8±2.2
Threshold (nA)	−0.0309±.0084	−.0291±.0075
Action Potential Initiation (mV)	−26.1±2.4	−25.6±2.8
Action Potential Duration (at 30% height, ms)	2.50±0.28	3.08±0.65
Action Potential Height (mV)	74.3±5.0	69.8±7.2
Input Resistance (MΩ)	1438.57±240.28	1302.85±239.85
Firing of Action Potentials (Phasic/Tonic)	17/3	9/2

Significant differences between cell types are determined by t-test (* = p<0.05).

There were no differences in the passive properties of neurons with and without an AHP (Table 1); cell capacitance, a determinate of cell size, also did not differ in these two types of neurons (23.24±3.16 pF in AHP +ve neurons vs. 16.08±1.973 pF in AHP −ve neurons), nor did input resistance (1.43±0.24 GΩ in AHP +ve neurons vs. 1.30±0.23 GΩ in AHP −ve neurons).

In 21 neurons, the average magnitude of the AHP was −7.53±0.72 mV, the duration of the initial phase of the AHP was 266.83±35.02 ms, and AHP τ value was 111.53±12.68 ms.

In voltage clamp experiments, the magnitude of inward and outward current differed in two types of mouse enteric neuron (Fig. 2C). In a representative voltage clamp tracing (Fig. 2C, inset), depolarization from a holding current of −60 mV to +10 mV resulted in fast inward currents followed by sustained outward current. The AHP +ve neurons had significantly larger current densities of both inward and outward currents. The peak inward current was −103.5±23.1 pA/pF in AHP +ve neurons vs. −43.6±13.9 pA/pF in AHP −ve neurons at +10 mV. The peak outward currents at the end of pulse was 117.7±22.9 pA/pF in AHP +ve neurons vs. 42.3±14.1 pA/pF in AHP-ve neurons at +50 mV. Significant differences, denoted with an asterisk, were determined via a two-way ANOVA analysis of both current density and voltage (p<0.05).

AHP +ve neurons display AHPs after both short and long pulse protocols (Fig. 3 A & B). Time derivatives of the APs show that in these neurons, there was no hump on the repolarization phase (Fig. 3 C & D).

### 3.3 Clotrimazole Blocks AHPs

**Figure 4:**
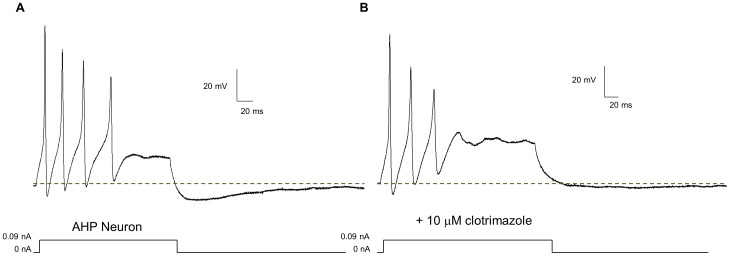
Clotrimazole block the after-hyperpolarization (AHP) and a portion of the sustained outward K^+^ current in AHP positive neurons. In neurons that show an AHP (A), Clotrimazole (10 µM), a putative blocker of the slow AHP [27], abolished the AHP (B).

Clotrimazole (10 uM), a putative blocker of intermediate conductance (IK) calcium-activated potassium channels [26] and the slow AHP [27], [28], was used to examine the AHP (Fig. 4). In 5 AHP-positive neurons, 10 µM clotrimazole completely ameliorated the AHP in agreement with *in situ* mouse enteric neurons recordings by Kunze et. al. (2006).

### 3.4 Tetrodotoxin Sensitivity in AHP-positive/negative Neurons

**Figure 5:**
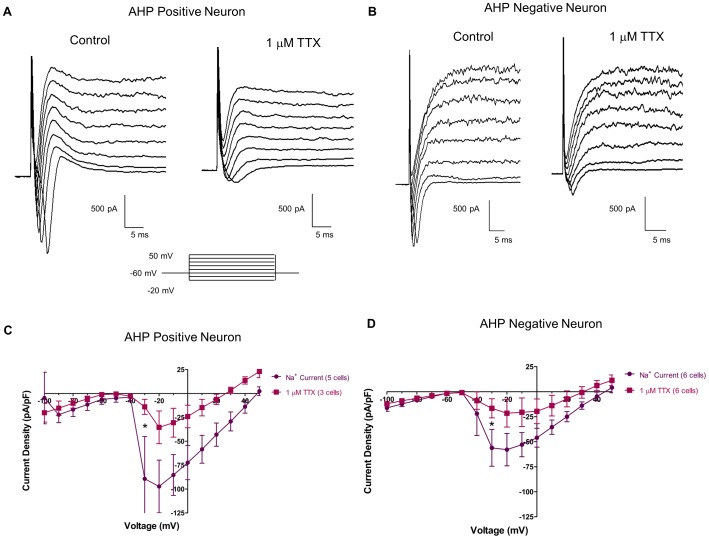
Tetrodotoxin (TTX) sensitive and TTX resistant Na^+^ channels are present in neurons with and without an after-hyperpolarization (AHP). Raw tracings of both AHP +ve neurons (A) and AHP -ve neurons (B) show a decrease in Na^+^ current in response to 1 µM TTX. Current density – voltage relationships showed that TTX (1 µM) significantly reduced peak Na^+^ current in both AHP +ve (C) and AHP −ve (D) neurons. Furthermore, both AHP +ve (C) and AHP −ve (D) neurons have some TTX resistant Na^+^ current channels that have a peak current density of ∼25 pA/pF in both cell types. Asterisks denote significance via two-way ANOVA of current density and voltage (p<0.05).

In both types of neurons, the inward currents were significantly inhibited by tetrodotoxin (TTX), a potent and selective inhibitor of certain Na^+^ channels (Narahashi et. al., 1964). The raw traces of both AHP +ve neurons (Fig. 5A), and AHP −ve (Fig. 5B) neurons show an attenuation of Na^+^ current in the presence of 1 µM TTX. Current density-voltage analysis (Fig. 5C & 5D) revealed that TTX significantly abolished some, but not all, of Na^+^ current in both cell types. In AHP +ve neurons, peak Na^+^ current at −30 mV was reduced from −89.2±44.2 pA/pF to −14.0±7.8 pA/pF. In AHP −ve neurons, at −30 mV, peak Na^+^ current was reduced from −56.1±18.4 pA/pF to −16.6±9.5 pA/pF. Asterisks denote significance via two-way ANOVA of current density and voltage (p<0.05).

### 3.5 Inward Current is Na^+^

**Figure 6:**
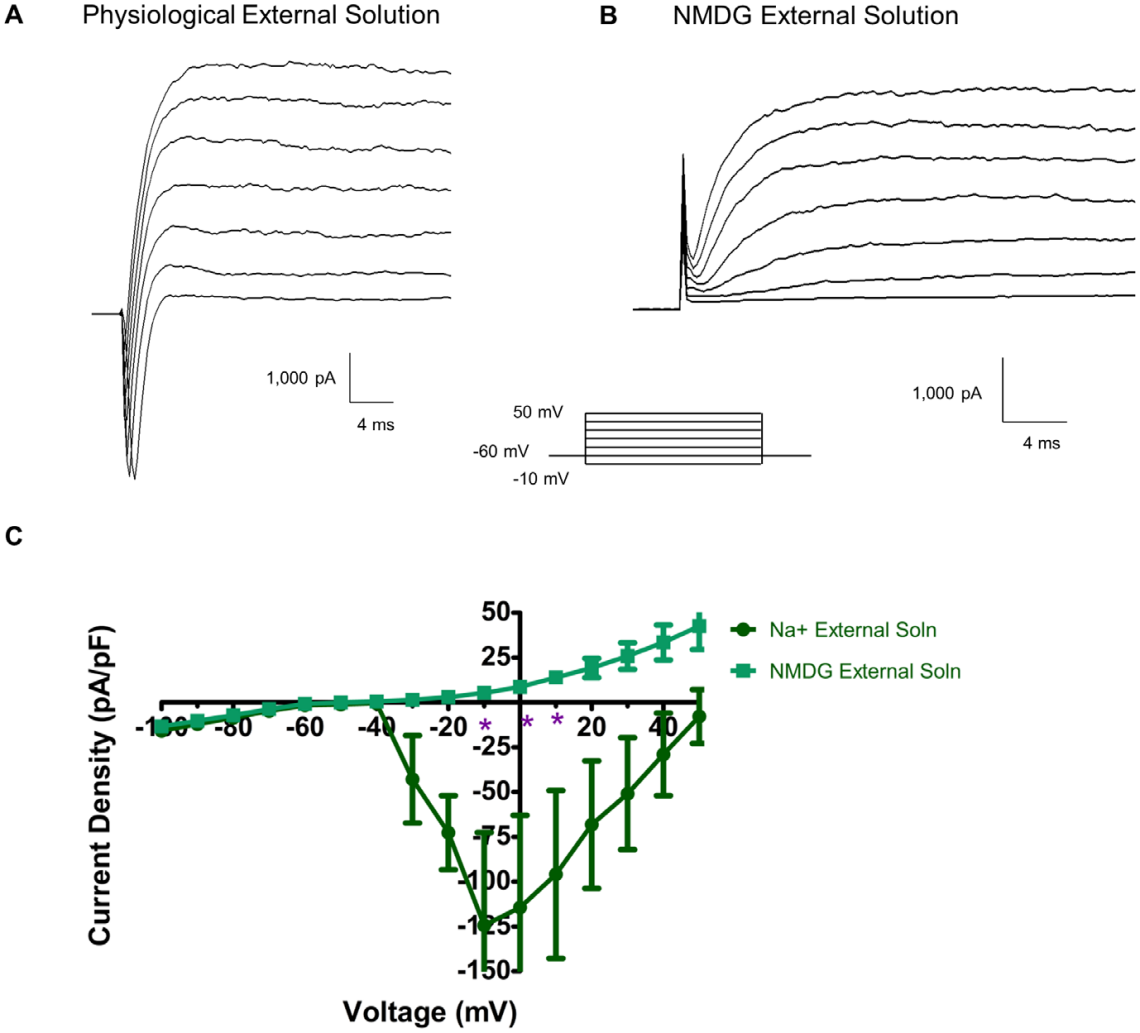
All inward current is Na^+^ current. Voltage clamp recordings made in the presence of Na^+^-containing physiological external solution (A) display quick inward (downward) Na^+^ currents. When the external solution is changed to Na^+^-free NMDG external solution (B), all apparent Na^+^ current is abolished. Current density-voltage relationship (C) shows the amelioration of Na^+^ current when the external solution is changed from Na^+^-containing to Na^+^-free solution.

Na^+^ was replaced with NMDG in the physiological external solution to determine the ionic basis of inward currents measured in the whole cell patch clamp recording. Fig. 6 shows that in physiological external solution with a high K^+^ internal solution, robust Na^+^ currents were seen in response to depolarization; at −10 mV the amplitude of Na^+^ current is −124.3±51.7 pA/pF. (Fig. 6A). Removing the Na^+^ from the external solution reduced the current to 5.2±1.2 pA/pF at −10 mV. Therefore, all inward current was due to Na^+^ ion movement.

The above studies reveal that primary cultures of adult mouse enteric neurons are functionally viable with robust action potentials, presence of afterhyperpolarizations, expression of calcium-binding proteins, and significant expression of TTX-sensitive and TTX-insensitive Na^+^ current. Many, but not all, of these findings are similar to those obtained from primary cultures of guinea-pig neurons, and in-situ recordings obtained with high resistance microelectrodes. In the next series of experiments, we determined the effect of morphine on isolated mouse enteric neurons.

### 3.6 Morphine Decreases Neuronal Excitability

**Figure 7:**
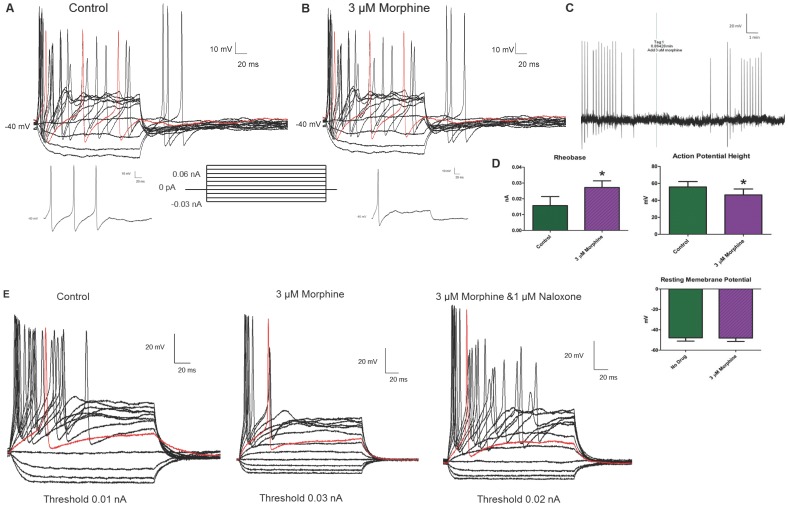
Morphine decreases neuronal excitability in enteric AHP +ve neurons without changing the resting membrane potential. In current clamp mode, AHP +ve neurons fire multiple, robust action potentials in the absence of drug (A). In the presence of 3 µM morphine (B), the height of the initial action potentials is reduced and the ability of the neurons to fire a second action potential is blocked. Furthermore, at the initial threshold of the cell the neuron is no longer able to fire in the presence of morphine (B), indicating a rise in the threshold. The threshold of the neuron is displayed as an inset below A and B; for this neuron the threshold is doubled from 0.01 nA to 0.02 nA. Anode break action potentials are seen in both tracings (A & B). A gap-free recording (C), which contains the current clamp protocol in (A), the addition of 3 µM morphine at the tag, a five minute waiting period and the current clamp protocol in (B) indicates that the resting membrane potential (solid black line) of the neuron does not change. Statistical analysis (D) indicates that morphine (3 µM) causes a significant increase in threshold and a decrease in action potential height (taken at 30% height at 0.09 nA current injection) while leaving the resting membrane potential unaffected (* = p<0.05 via t-test). n = 6 for all analyses. The reduction in excitability due to morphine is blocked by the μ opioid receptor antagonist naloxone (E).

Morphine significantly reduced neural excitability in AHP +ve neurons. Current clamp recordings of a representative AHP +ve neuron (Fig. 7A) showed that in the absence of drug, the neuron fires multiple, robust action potentials at a threshold of 0.01 nA, (insert below A) and higher current stimuli. Also, strong anode-break action potentials were obtained as a result of the removal of negative, hyperpolarizing current injections. The addition of 3 µM morphine (Fig. 7B) increased the threshold of the neuron (0.02 nA, insert below B), and altered the firing pattern from multiple to a single AP. In all cells tested, morphine significantly increased the threshold from an average baseline value of 0.010±0.001 nA to 0.026±0.004 nA. Furthermore, the height of the initial AP was significantly attenuated; initial AP height was 99.6±19.2 mV before morphine and 80.6±27.7 mV after morphine. Also of note is that morphine-treated neurons were no longer able to fire multiple action potentials even when the current stimulus was doubled. Interestingly, the height of the anode-break action potentials in the presence of morphine was unchanged. This reduction in neural excitability was not due to a change in the resting membrane potential (RMP). Figure 7C shows a gap-free recording of the same cell as in Fig. 7A & B, where the addition of 3 µM morphine did not alter the RMP, but reduced the subsequent action potentials. The differences in the threshold, action potential height, and RMP are presented in the bar graphs (Figure 7D). Input resistance was not altered by morphine (1.26±0.47 GΩ for control vs. 0.93±0.38 GΩ in the presence of 3 µM morphine). APs were initiated at the same voltage before and after morphine (−46.0±7.5 mV for control vs. −42.6±8.8 mV after 3 µM morphine) Significance was determined using t-test analysis of 6 neurons (p<0.05). The reduction in excitability caused by 3 µM morphine was reversed in the presence of 1 µM naloxone (Fig. 7E).

### 3.7 Morphine Inhibits Na^+^ Current

**Figure 8:**
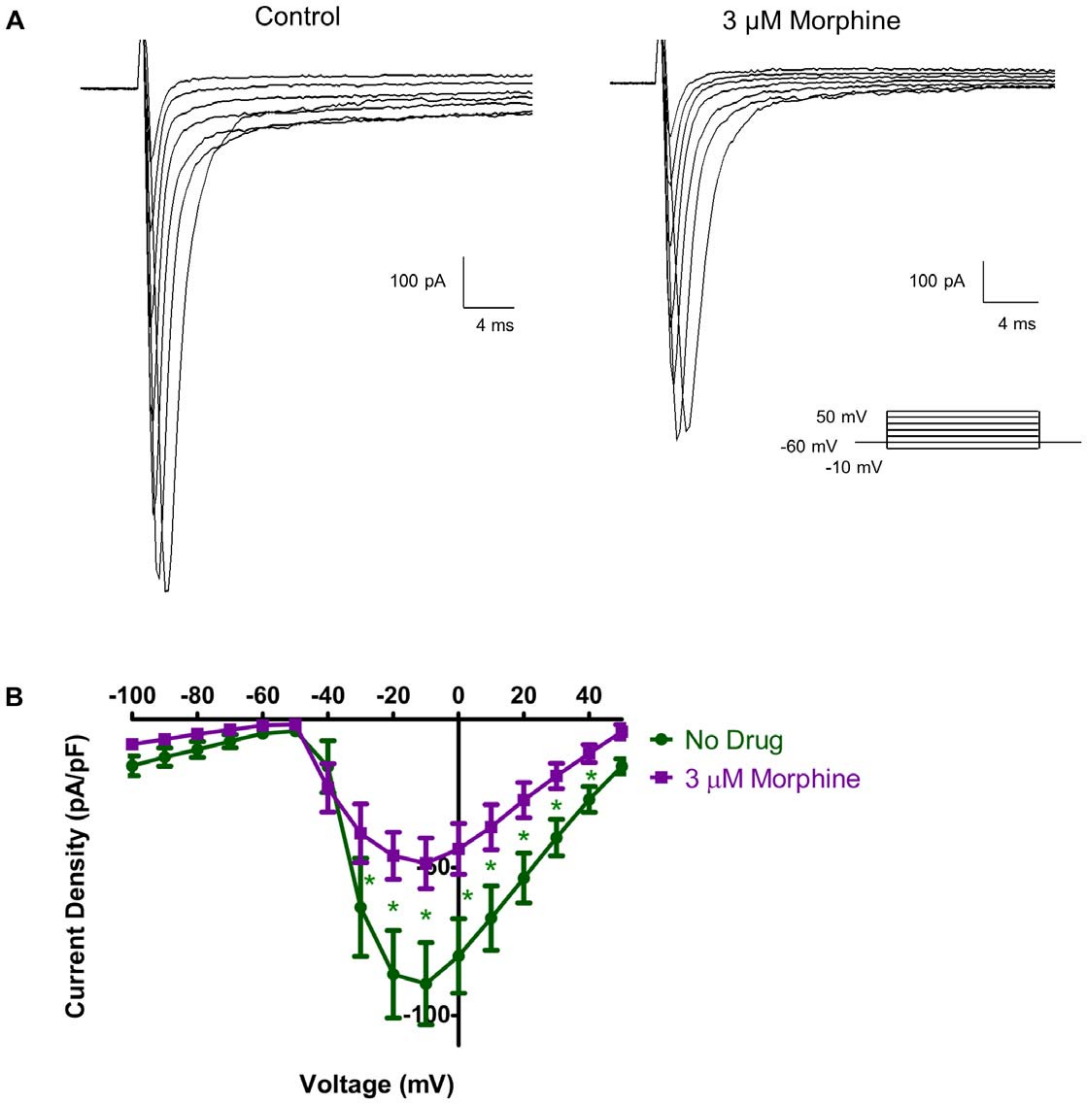
Morphine inhibits Na^+^ channel current. During voltage clamp recordings (A), Na^+^ channel current is seen as a quick inward spike in response to depolarizing steps in absence of drug (A, left panel). Cs^+^ has been added to the internal solution to block all confounding K^+^ current. In the presence of 3 µM morphine (A, right panel), the amplitude of Na^+^ channel currents is reduced. A current density-voltage relationship (B) shows that morphine (3 µM) significantly inhibits Na^+^ flow. Significance is determined via two-way ANOVA (n = 8, * = p<0.05).

To further examine the effects of morphine on Na^+^ currents, a Cs^+^-containing internal solution was used to block all K^+^ currents. In the presence of Cs^+^, distinct inward Na^+^ currents were seen in response to depolarizing steps in voltage clamp mode (Fig. 8A, left panel). At −10 mV, the Na^+^ current density in physiological external solution was −89.2±13.8 pA/pF. In the presence of 3 µM morphine, these currents were noticeably decreased to −48.5±8.5 pA/pF (Figure 8A, right panel). Statistical analysis of current density–voltage relationships showed that morphine significantly attenuated Na^+^ current via matched two-way ANOVA analysis of current density and voltage in 8 neurons (p<0.05).

### 3.8 Morphine Inactivates Na^+^ Current

**Figure 9:**
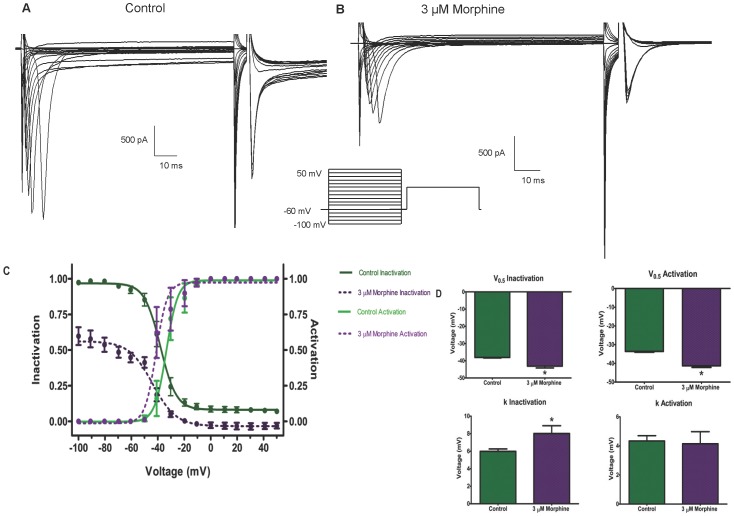
Morphine alters the inactivation and activation kinetics of Na^+^ channels. Na^+^ channel inactivation was examined using a double-pulse protocol without drug (A) and in the presence of 3 µM morphine (B). Boltzmann curve analysis (C) of inactivation and activation kinetic of Na^+^ indicate a leftward shift for both curves in response to morphine, indicated by a significant difference in V_0.5_ values (D). The inactivation curve also experienced a downward shift due to morphine, indicated by a significant difference in k (D).

The voltage dependence of steady-state inactivation kinetics and the effects of morphine were studied using a double-pulse protocol (Fig. 9 A & B). A variable conditioning pulse was applied from −100 mV to +50 mV in 10 mV steps for 50 ms. The test potential was 0 mV. For a conventional time- and voltage-dependent Hodgkin-Huxley conductance, the steady-state inactivation curve describes the relative number of available Na^+^ channels as a function of voltage. The resultant sigmoidal curve was fit by a Boltzman distribution (Fig. 9C).

In the absence of morphine, V_0.5_, the voltage at which half the channels are inactivated, was −38.0±0.3 mV with a slope factor of 5.9±0.2 mV (Fig. 9D). A small fraction of Na^+^ channels remained available at more positive conditioning potentials. In the presence of morphine, two major differences were observed. First, the V_0.5_ shifted to −43.1±1.0 mV with a slope factor of 8.0±0.8 mV and secondly there was a significant decrease in the availability of the channels at all potentials. At a conditioning potential of −100 mV, the fraction of available channels was approximately 50% in the presence of morphine. At more positive conditioning potentials, almost all channels were inactivated.

Morphine also affected the activation kinetics of the Na^+^ channels (Fig. 9C). Morphine induced a leftward shift of the Na^+^ activation curve, as indicated by a significant increase in the V_0.5_ of activation. The V_0.5_ increased from −33.6±0.4 mV in the absence of drug to −41.3±0.8 mV in the presence of morphine (Fig. 8D). The slope factor was not significantly affected (4.3±0.3 without drug, 4.1±0.8 with morphine).

### 3.9 Morphine Inactivates TTX-resistant Na^+^ Channels

**Figure 10:**
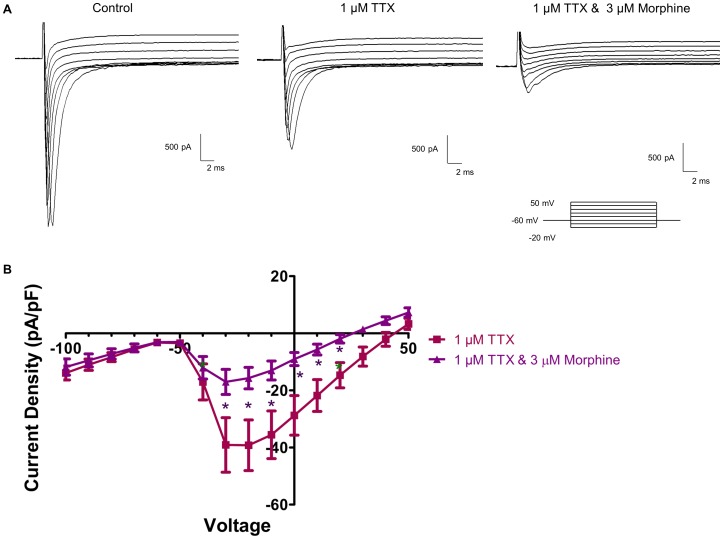
Morphine Inhibits TTX-resistant Na^+^ channels. TTX-resistant Na^+^ current was examined in the presence of Cs^+^-containing internal solution (A). 1 µM TTX blocked all TTX-sensitive Na^+^ channels (A, middle panel), and then morphine (3 µM, A, right panel) was added to assess the effects of the drug on TTX-resistant Na^+^ current. Current density- voltage analysis (B) showed that TTX-resistance Na^+^ current is partially inhibited by the addition of 3 µM morphine.

**Figure 11:**
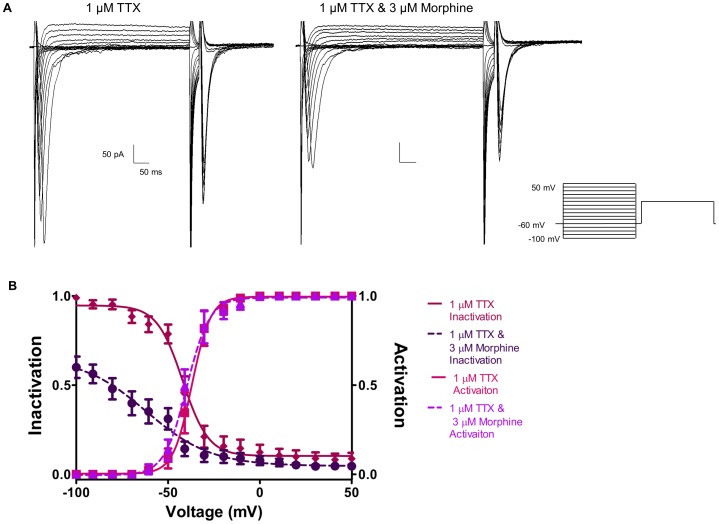
Morphine alters the inactivation kinetics of TTX-resistant Na^+^ channels. A double-pulse inactivation protocol was used to evaluate the inactivation kinetics of Na+ current in the presence of 1 µM TTX (A) and 1 µM TTX with 3 µM morphine (B). Boltzmann analysis (B) reveled that morphine shifted the inactivation curve of TTX-resistant Na^+^ channels down and to the left, as reflected in a significant reduction of the v_0.5_ and k values. Activation kinetics were unaffected.

To further determine if morphine inhibits and inactivates TTX-resistant Na^+^ currents, Na^+^ current density and voltage-dependence of steady-state inactivation and activation were repeated in the presence of TTX to block all TTX-sensitive Na^+^ channels (Fig. 10 & 11). Fig. 10A shows that the addition of 1 µM TTX blocked a large portion of the inward Na^+^ current, which was further reduced by 3 µM morphine. Morphine significantly reduced Na^+^ current in the presence of TTX from −39.1±9.5 pA/pF to −13.0±3.31 pA/pF at −10 mV (Fig. 10B). The same inactivation protocol used to generate the data in Fig. 8 was repeated for the data in Fig. 11. Similar to the effects on I_Na_, morphine shifted the voltage-dependent inactivation curve in the presence of TTX (Fig. 11B) and markedly reduced the fraction of channel availability at negative potentials. However, the kinetics of activation were not significantly altered by morphine in the presence of TTX.

### 3.10 Morphine Tolerance in a Single Neuron

**Figure 12:**
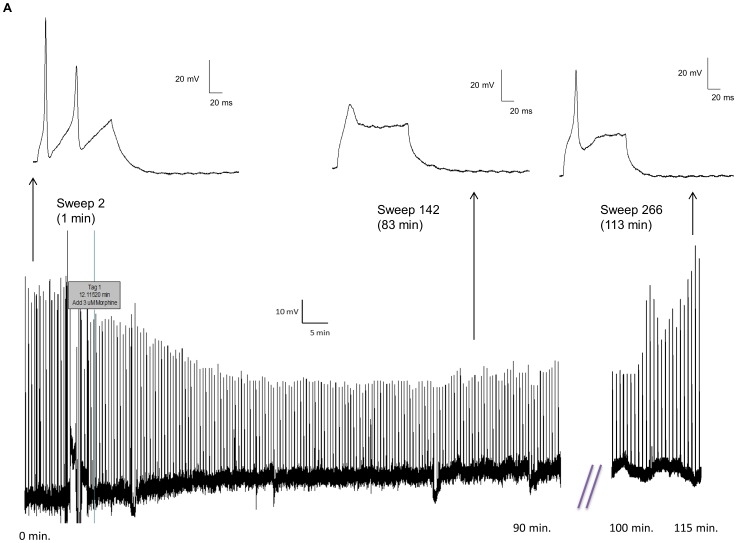
Morphine causes tolerance within a single mouse enteric neuron. (A) A gap-free tracing shows an enteric neuron that fires multiple action potentials (above inset, left) in response to a 0.05 nA of current applied for 100 ms every 30 sec. The addition of morphine indicated at the tag results in a decrease in action potential amplitude and the inhibition of subsequent action potentials. The action potential is virtually non-existent after 83 min (above insert, middle). After nearly two hours, the action potential increases in amplitude, indicating a recovery from the effects of morphine (113 min., above insert, right). This recovery is possible without a significant change in the resting membrane potential.

One of the hallmark features of morphine's effects is the development of tolerance upon repeated or prolonged exposure. We have previously demonstrated that 2 hour morphine incubation resulted in tolerance in the mouse ileum [29]. To further examine whether tolerance could be rendered in a single enteric neuron, a continuous recording was obtained of action potentials elicited by 0.05 nA of current for 100 ms every 30 sec (Fig. 12). In 3 cells that could be maintained over 2 hours, morphine initially reduced the action potential height and multiple spikes, which returned towards baseline values after 2 hours. The example of a raw tracing shown in Fig. 12 demonstrates that before morphine was added, the neuron fired multiple action potentials (1.5 min., above left inset). The addition of 3 µM morphine, indicated by the tag in the gap-free recording, shows that morphine decreased AP height and eventually ameliorated APs after 83 min. (above middle inset). After nearly 2 hours of recordings, APs return and increase in height, although the neuron continued to fire single APs (113 min., above right insert). Morphine is stable throughout the experiment as subsequent cells tested with the same solution showed a full response to the effects of morphine (data not shown).

## Discussion

Our findings characterize adult isolated mouse enteric neurons and establish a novel mechanism for the effects of morphine in the gastrointestinal tract. Previous studies have mainly focused on the guinea pig enteric neurons, both from electrophysiological and immunohistochemical coding perspectives. Defining a murine model is key to using genetically altered animals in future experiments. Additionally, our data illustrate a potential mechanism by which morphine alters enteric neuronal excitability.

Unlike the guinea pig, the myenteric plexus in the mouse adheres strongly to both longitudinal and circular muscle layers [30]. This was confirmed by whole mount staining with β-III tubulin of both layers. In this study, we chose to isolate neurons from the longitudinal muscle to reduce the contamination from fecal contents that occur when the circular muscle layers were used. A large proportion of cells isolated using this methodology were neurons expressing calbindin and calretenin positive staining. Calretinin positive neurons in both guinea pigs and mouse are suggested to co-localize with choline acetyltransferase (ChAT) and tachykinin (TK) but not nitric oxide synthase (NOS) consistent with being excitatory longitudinal muscle motor neurons and ascending interneurons [11], [31] while calbindin has been suggested to be associated with myenteric intrinsic primary afferent neurons. In addition, our mixed culture also contained enteric glia, which may be important for neuronal cell culture survival [32]. The cultured neurons were difficult to morphologically distinguish into Types I and II neurons as previously shown in whole-mount preparations [31].

About two-thirds of the neurons demonstrated an afterhyperpolarization (AHP). Some distinguishing characteristics exist between the AHP described here and in the literature. In the earlier studies examining AHP duration in guinea pig whole tissue LMMP preparations, AHPs typically lasted 10 s [3]. In *in situ* mouse studies, this duration was considerably shorter, less than 2 s in most cells [33]. Our studies report an even shorter AHP duration; with an average initial phase of 266 ms. A possible reason for this difference in the AHP duration may be due to the recordings being made in isolated single cells as opposed to whole-tissue microelectode or in-situ recordings, where synaptic connections may result in prolonged duration of the voltage changes. It is also possible that the difference in the AHP duration may be the result of differences in the electrophysiological properties of the neurons due to the culturing process, or the whole cell recoding conditions used in this study.

Previous studies from guinea pig and in-situ recordings from mouse myenteric plexus have characterized myenteric neurons containing an AHP as AH (sensory) as opposed to S (motor) neurons [4], [7]. AH neurons typically demonstrate a hump in the downward phase of the action potential, although this was not observed in the current study, as indicated by the lack of a hump in the time derivative of the action potential. The AHP was abolished by the calcium-activated potassium channel blocker, clotrimazole [27]. In addition to the presence of an AHP, the two subtypes also differed in the density of sodium channels and the resting membrane potential. In both subtypes, prominent TTX-resistant sodium currents with similar densities were observed, however total sodium channel currents were greater in the neurons with an AHP. Previous studies have suggested the presence of TTX-resistant sodium currents in both guinea pig and mouse AH neurons [5], [33] which was confirmed in our cultured neurons. Since both subtypes demonstrate TTX-resistant sodium channels and multiple action potentials with the AHP being the only distinguishing feature, it is not possible at the present time to define the subtypes as AH and S neurons, since primary afferent neurons without AHP have also been recorded in the mouse large intestine and pig [10], [34]. Nevertheless, the immunohistochemical coding (calretinin and calbindin) and electrophysiological differences suggest the presence of at least two subtypes of neurons in the mouse myenteric plexus cultures. The presence of an AHP and TTX-resistant Na^+^ currents would suggest the presence of AH neurons in our culture in agreement with other studies of enteric neurons in the mouse [10], [33]. However, our recordings do not demonstrate a “hump” on the repolarization phase of the AP or hyperpolarization-activated current (I_h_) [33]. One explanation for these differences may be the strain and/or sex of mouse used. Nurgali et. al. (2004) [10] utilized BALBc mice and Mao et. al. (2006) [33] used female C57BL/6 mice. Our current studies were performed in male Swiss Webster mice, because our previous studies have shown significant morphine effects in these animals [35], [36].

The constipating effects of morphine are well established; reduced peristalsis, increased water and electrolyte absorption, and antisecretory actions [15] are attributed to actions on the enteric neurons which reduce action potentials both pre- and post-synaptically [21], [37]. This study demonstrates that morphine decreased neural excitability in AH neurons via an increase in threshold, a reduction in action potential height, as well as inhibition of the ability of the neuron to fire multiple action potentials. Notably, this decrease in excitability was not accompanied by a change in the resting membrane potential of the neuron. This is an extremely important observation, as previous studies in the brain and the gut have attributed the decrease in neurotransmitter release to hyperpolarization of neurons as a result of potassium channel activation [21], [22], [38]. The 3 µM morphine dose chosen for this study was based on our previous findings demonstrating functional inhibition of EFS-induced contraction in mouse and guinea pig ileum [29], [36] At this dose, the input resistance of the myenteric neurons was unchanged. Similar effects were also observed in a few cells examined at 10 µM morphine dose (data not shown).

Previous studies have largely attributed the reduced neuronal excitability seen in these neurons to an increase in K^+^ conductance [21], [37], [39]. Additionally, this increase in K^+^ conductance is important for the effects of opioids in central nervous system locus coeruleus neurons [38], [40]. Indeed, this may be an influential mechanism in addition to the effect on Na^+^ current seen by morphine in the gut. In our experiments, every attempt was made to isolate the effects of Na^+^ from K^+^ by using an intracellular solution containing Cs to block K^+^ channels. In the previous work that examined the effects of opioids on specifically AH (type 2) cells of the guinea pig myenteric neurons, only about 10% of these cells responded with a modest hyperpolarization of 2–10 mV in response to normorphine. This hyperpolarization declined after 1–2 minutes and subsequent normorphine applications often had no effect. This suggest that although the increase in K^+^ conductance may be an important mechanism for the immediate reduction in decreased myenteric neuron excitability, it may not be the sole mechanism or a mechanism sufficient in itself. It is possible that both the K^+^ and the Na^+^ mechanisms are important for the reduced excitability in response to opioids. This observation led us to take a closer examination of the direct effects of morphine on Na^+^ channel function. Furthermore, the reduced excitability attributed to morphine is a receptor-mediated event, as this reduction in excitability can be reversed by naloxone. This study examines the effects of morphine on AHP positive neurons of the ileal LMMP preparation. The limitation of this study is that these effects cannot necessarily be extrapolated to all AHP positive neurons in the ENS, such as those found in other plexii or more intimately connected to the circular muscle, nor to neurons that lack an AHP.

Immunohistochemical verification of μ opioid receptors was not possible as the antibodies tested did not pass appropriate controls. This is not surprising, as a recent study examined six commercially available antibodies, including the ones used here, and found that they were all not specific to the μ opioid receptor [25]. However future studies may utilize a μ-opioid receptor specific blocker such as cyprodime, to confirm the presence of the μ-receptor [41].

**Figure 13:**
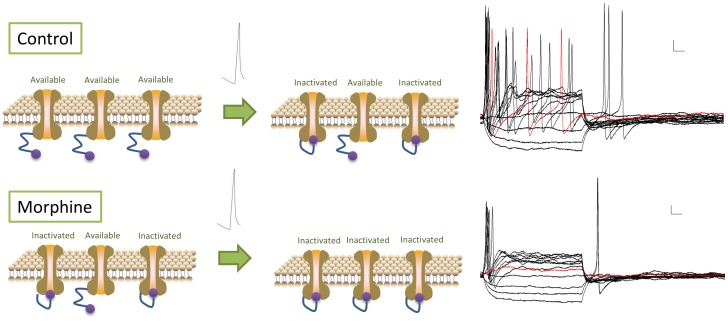
Morphine inhibits multiple action potentials by inactivating Na^+^ channels. Under drug-free conditions (top panel), before a stimulus is applied all Na^+^ channels are available for activation. After the first action potential, some Na^+^ channels become inactivated, leading to blunted subsequent action potentials. In the presence of 3 µM morphine (bottom panel), some Na^+^ channels are inactivated before a stimulus is applied, resulted in a truncated initial action potential. After the first action potential, all Na^+^ channels become inactivated, resulting in a blockade of any consecutive action potentials.

We found that morphine acutely inhibits the amplitude of Na^+^ current and determined the mechanism of this by examining the inactivation and activation kinetics of the Na^+^ channel in response to morphine. The plateau of the Na^+^ channel inactivation curve was significantly depressed in the presence of morphine. At negative voltages (below −70 mV), acute exposure to morphine resulted in nearly half of the Na^+^ channels being inactivated. Na^+^ channel inactivation is achieved by the closure of an inactivation gate formed by the intracellular loop between domains III and IV [42]. μ Opioid receptor activation may activate intracellular second messenger signaling molecules which bind to the intracellular loop and favor Na^+^ channel inactivation. Interestingly, in the absence of morphine, TTX-sensitive sodium channels remained available at positive voltages, indicating that the overall population of Na^+^ channels is resistant to total inactivation under normal conditions. In the presence of morphine, a large proportion of the Na+ channels are inactivated at positive potentials, suggesting that morphine leads to total Na^+^ channel inactivation. Conceptually, this can be examined by observing the voltage at which action potentials are generated. In AH +ve neurons, the initiation of action potentials occurs at −31.1±2.4 mV. At this point in the inactivation Boltzmann curve, without drug present, a certain portion of the Na^+^ channels remain available for activation and can trigger a second action potential. However, in the presence of morphine, all of the Na^+^ channels were shifted to an inactivated state. Thus, after Na^+^ channels are activated during the first action potential, recovery from inactivation may be reduced, resulting in the inability to fire multiple spikes (Fig. 13). The effects of morphine remain evident in the presence of TTX, indicating that these effects occur, at least partially, in TTX-resistant sodium channels. Direct observations in genetically altered animals, such as Nav1.8-null mice, would be needed to confirm these conclusions.

We have previously shown that chronic morphine *in vivo* leads to enhanced excitability of dorsal root ganglion neurons due to a shift in the voltage-dependence of activation of TTX-resistant sodium currents resulting in a larger window of channel availability [35]. These differences between chronic exposure inducing hypernociception in sensory neurons, and acute morphine-induced decreased excitability of enteric neurons are consistent with the notion that opioid-induced bowel dysfunction is a complex phenomenon with decreased peristalsis (involving enteric neurons) and abdominal pain (involving sensory neurons) that are separate entities.

We also investigated whether our model could be used to study morphine tolerance in a single enteric neuron. Enteric neurons demonstrated reduced neuronal excitability in the continued presence of morphine. After nearly two hours the excitability of the neurons began to recover, indicative of tolerance development, as seen in isolated organ studies [29]. This demonstrates that our current acute data are not an artifact of Na^+^ channel run-down. Future studies will address the mechanism in which tolerance develops in this model.

In conclusion, the results of these experiments suggest that reduction in Na^+^ current, rather than alterations in K^+^ or Ca^2+^ currents studied previously, are the main driving force underlying the reduced neuronal excitability seen in response to morphine in enteric neurons. The model we have developed will prove highly valuable in that it allows the characterization of cultured murine enteric neurons, but also provides a mechanism to study tolerance in a single enteric neuron. If we assume that the AHP positive neurons in this study are intrinsic sensory neurons, this may contribute to reduced peristalsis in response to morphine by interrupting the neuronal circuit required for proper defecation. Sensory neurons are required to activate the neural pathways that control both oral contraction and anal relaxation relative to the sensed food bolus [43]. By interrupting this neural pathway, normal gut peristalsis is altered. Further study is required to determine the exact mechanism behind this disruption.
