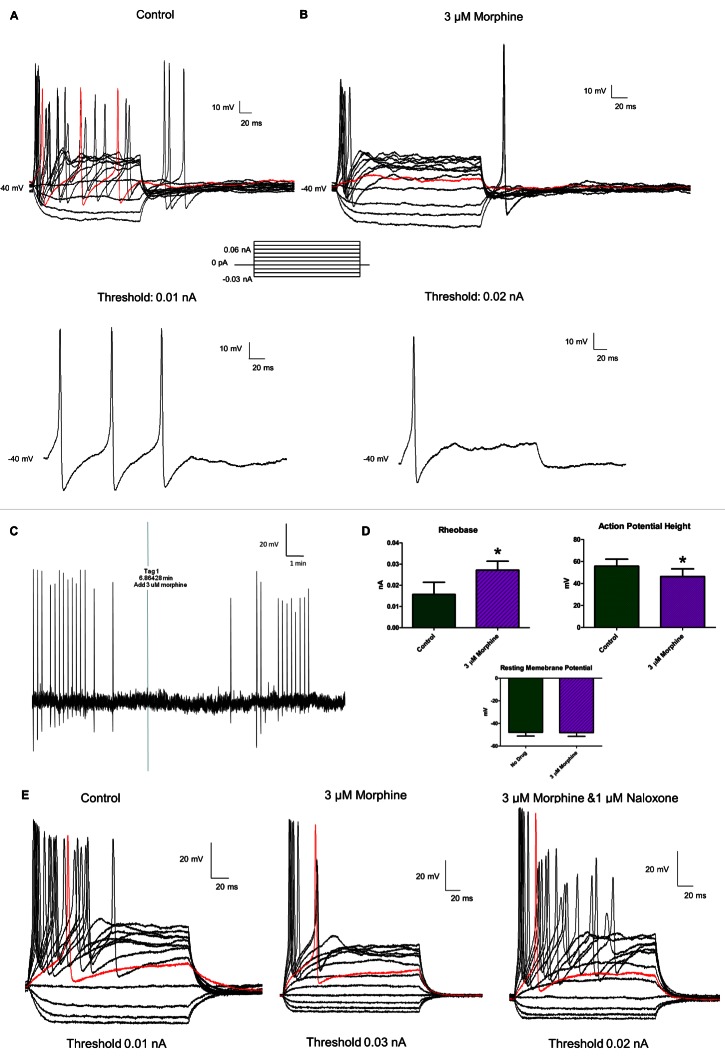# Correction: Morphine Decreases Enteric Neuron Excitability via Inhibition of Sodium Channels

**DOI:** 10.1371/annotation/c2d0d839-3fea-4dfd-ad78-4e4fb1271b20

**Published:** 2013-05-23

**Authors:** Tricia H. Smith, John R. Grider, William L. Dewey, Hamid I. Akbarali

There was an error in Figure 7. The top trace in part B was a duplicate of part A. Please use the following link to access the correct version of Figure 7: 

**Figure:**